# Inflammatory Bowel Disease and Its Association With Perinuclear Antineutrophil Cytoplasmic Antibodies: A Systematic Review

**DOI:** 10.7759/cureus.57872

**Published:** 2024-04-08

**Authors:** Harleen Kaur, Malik Kasapoglu, Rajesh Yadavalli, Sarosh Nawaz, Abdulaziz Althwanay, Esraa M AlEdani, Ann Kashmer Yu

**Affiliations:** 1 Department of Internal Medicine, California Institute of Behavioral Neurosciences & Psychology, Fairfield, USA; 2 Department of Internal Medicine, Rajiv Gandhi Institute of Medical Sciences, Adilabad, IND; 3 Department of Psychiatry, California Institute of Behavioral Neurosciences & Psychology, Fairfield, USA; 4 Department of Dermatology, California Institute of Behavioral Neurosciences & Psychology, Fairfield, USA; 5 College of Medicine, Imam Abdulrahman Bin Faisal University, Dammam, SAU; 6 Department of Dermatology and Internal Medicine, California Institute of Behavioral Neurosciences & Psychology, Fairfield, USA

**Keywords:** autoimmune colitis, crohn's disease (cd), gastrointestinal disease, inflammatory bowel disease, perinuclear antineutrophil cytoplasmic antibody, ulcerative colitis (uc)

## Abstract

An idiopathic condition known as inflammatory bowel disease (IBD) is characterized by a dysregulated immune response to the intestinal flora of the host. It falls into one of two primary categories: ulcerative colitis or Crohn's disease. A wide range of disorders, both clinically and genetically, can cause IBD. The purpose of this thorough analysis is to determine the significance and reliability of the correlation between perinuclear antineutrophil cytoplasmic antibodies (p-ANCA) and IBD, as well as the implications of this correlation for the diagnosis and treatment of IBD. Ten pertinent studies were identified from a starting pool of 20 articles in this systematic review, which was conducted in accordance with the Preferred Reporting Items for Systematic Reviews and Meta-Analyses (PRISMA) 2020 guidelines. These studies addressed treatment, complications, limitations, and outcome in addition to the presence or lack of p-ANCA in patients with IBD. In conclusion, p-ANCA is more strongly linked to inflammatory bowel illness than Crohn's disease, primarily ulcerative colitis. Some evidence suggests that there is a decrease in p-ANCA to some extent with medical or surgical interventions, but the exact intervention is not yet clear. There is less evidence suggesting that the medical or surgical treatments used in patients with IBD cause an increase or decrease in p-ANCA.

## Introduction and background

A general name for recurrent, chronic inflammatory bowel illnesses is inflammatory bowel disease (IBD). Crohn's disease (CD) and ulcerative colitis (UC) are the two primary subgroups [1]. A wide range of disorders, both clinically and genetically, can cause IBD [2]. Certain antibodies against microbial and host antigens are linked to IBD [3]. These antibodies may help with the differential diagnosis and pathogenetic understanding of IBD. Given that IBD is a chronic, lifelong condition that often manifests at an early age, a significant rise in its prevalence is anticipated over the next several decades [4]. Appropriate subtype classification is essential for effective management. However, because of unusual appearances, it can occasionally be difficult to determine the accurate diagnosis. In many patients, even with a comprehensive work-up that includes imaging of the small bowel, esophagogastroduodenoscopy, ileocolonoscopy with histology, and physical and laboratory examinations, a classification cannot be made, or patients must be reclassified as the disease progresses [1]. To effectively assess the level of disease activity, an endoscopic evaluation is preferred; however, performing frequent endoscopic examinations is challenging due to substantial financial and physical demands [5]. Nonetheless, it has been noted that the hazards associated with invasive diagnostic procedures like endoscopy and gastrointestinal series can be avoided by using disease-specific serologic markers for UC and CD [6].

Based on preliminary information, it appears that IBD has an asymptomatic preclinical phase, similar to other immune-mediated disorders. Understanding this stage might make it possible to forecast diseases. Using preclinical serum samples kept in the US Department of Defense Serum Repository (DoDSR), the PREDICTS (Proteomic Evaluation and Discovery in an IBD Cohort of Tri-Service Subjects) project was started with the intention of finding biomarkers linked to the onset of IBD [4]. Antineutrophil cytoplasmic antibody (ANCA), which is directed against proteinase 3 (PR3), is a marker for granulomatosis with polyangiitis and is also seen in patients with IBD, particularly UC [7]. The serologic markers that have been identified as diagnostic markers for CD include anti-*Saccharomyces cerevisiae* antibodies (ASCAs), anti-*Pseudomonas fluorescens*-associated sequence I2 antibodies, anti-*Escherichia coli* outer membrane porin C antibodies, anti-bacterial flagellin antibodies, and anti-CD peptide antibodies [6]. The two antibodies in IBD that have been studied the most are ANCA and ASCA. ANCA can be classified as follows based on how they stain in indirect immunofluorescence (IIF): p-ANCA has perinuclear fluorescence, c-ANCA has cytoplasmic fluorescence, and x-ANCA (also known as "atypical ANCA") has rim-like perinuclear fluorescence. The term "perinuclear" refers to the rim-like staining of p-ANCA [1]. ANCAs were identified in the 1980s as a useful marker for systemic vasculitis, such as microscopic polyangiitis (MPA) or granulomatosis-associated polyangiitis (GPA). In the 1990s, it was discovered that a sizable portion of individuals with IBD had positive ANCA on IIF. Bossuyt's review indicated that patients with UC experienced this more frequently than those with CD. ASCA was also found to be highly specific for CD [8]. Contrarily, perinuclear antineutrophil cytoplasmic antibody (p-ANCA), which has a prevalence of 60-80% compared to 10% in CD patients, was thought to be a relatively specific marker for UC [9]. As a result, it was conventionally taught that ANCA and ASCA together could be useful in differentiating between UC and CD [8].

For ANCA-associated vasculitis (AAV), ANCAs serve as diagnostic markers. Apart from AAV, ANCA positivity can also be seen in patients with different immunological conditions like primary biliary cholangitis and rheumatoid arthritis. p-ANCA positivity can be used as a diagnostic marker for UC and has also been reported in 10-80% of UC patients who do not have complicated AAV. PR3-ANCA positivity, which is equivalent to c-ANCA, has been shown to have a sensitivity of 58% and a specificity of 93% in the differential diagnosis of UC and CD [10]. It is also observable in UC patients. Antibodies directed against the surface antigens of bacteria or fungi are known as serological markers, and they can serve as indicators of pathological conditions, physiological processes, and treatment outcomes. They are measurable and evaluable in an objective manner [9]. Prior research has demonstrated that serum biomarkers are insufficient for determining the severity of an IBD patient's condition or predicting how well they will respond to treatment [6]. It wasn't until the 2000s that the differences between atypical and typical p-ANCA were consistently made. This is why studies carried out in the 1990s discovered that in IBD patients with UC, p-ANCA dominated the ANCA pattern; however, studies carried out in the 2000s discovered that in these individuals, atypical p-ANCA predominated [8].

In this systematic review, we aim to investigate the association of p-ANCA with IBD and see the strength and consistency of the association between p-ANCA and IBD and the implications of this association in the diagnosis and management of IBD.

## Review

Methods

A PICO (Population, Intervention, Comparison, and Outcome) was developed for this systematic review in order to offer a structured framework that would direct the methods section. The study's patient population (P) is made up of people with IBDs, such as those who have UC and CD. The primary focus of the investigation (I) is to evaluate the presence of or level of p-ANCA in IBD patients. This will be compared (C) to the IBD patients without p-ANCA or IBD patients with different levels of p-ANCA. The strength and consistency of the relationship between p-ANCA and IBD is the main outcome (O) that needs to be evaluated. This entails determining how much p-ANCA is present in IBD patients and whether it differs between CD and UC, two separate forms of the disease. The secondary outcome is to assess the implications of this association in the diagnosis and management of IBD. This includes aspects like diagnostic accuracy, disease severity, and treatment strategies influenced by p-ANCA.

In accordance with the most recent Preferred Reporting Items for Systematic Reviews and Meta-Analyses (PRISMA) statement 2020, this systematic review was conducted and reviewed [11]. A computerized search of the literature was done between January 2018 and January 2022.

Search Strategy

For this systemic review, only PubMed, PubMed Central (PMC), ScienceDirect, and Google Scholar were used as research databases and search engines. As indicated in Table 1, we combined the pertinent concepts with certain keywords using the Boolean terms "OR" and "AND."

**Table 1:** PubMed search strategy with regular keywords p-ANCA: perinuclear antineutrophil cytoplasmic antibody; ANCA: antineutrophil cytoplasmic antibody

Concepts	Keywords	PubMed Search Builder
p-ANCA	p ANCA, ANCA, perinuclear anti neutrophil cytoplasmic antibodies	p ANCA OR ANCA OR perinuclear anti neutrophil cytoplasmic antibodies
Inflammatory bowel disease	Inflammatory bowel disease	Inflammatory bowel disease

Similarly, the following Medical Subject Headings (MeSH) strategy was created using the same concepts as keywords. We chose subheadings such as immunology, physiopathology, and pathology. The results are shown in Table 2. The results for the advanced search strategy are shown in Table 3.

**Table 2:** MeSH strategy p-ANCA: perinuclear antineutrophil cytoplasmic antibody; MeSH: Medical Subject Headings

Keywords	MeSH strategy
Inflammatory bowel disease	"Inflammatory Bowel Diseases"[Mesh]
p-ANCA	(“Antibodies, Antineutrophil Cytoplasmic/immunology"[Majr] OR "Antibodies, Antineutrophil Cytoplasmic/physiology"[Majr] )

**Table 3:** Advanced search strategy

Advanced search strategy
(("Inflammatory Bowel Diseases"[Mesh])) AND ( "Antibodies, Antineutrophil Cytoplasmic/immunology"[Majr] OR "Antibodies, Antineutrophil Cytoplasmic/physiology"[Majr] )

Screening of Articles

All the relevant articles were collected and duplicates were eliminated. Next, based on the title, abstract, and full-text reading, the pertinent articles were filtered out. Ultimately, a selection of 11 articles underwent quality evaluation tools.

Inclusion Criteria

The paper focuses on studies involving patients of all age groups who have been diagnosed with IBD, including CD and UC. It also includes studies that have assessed the presence and levels of p-ANCA in IBD patients. Included as well are studies that provide clinical data on IBD patients, including disease severity, treatment regimens, and outcomes. Cohort studies, non-randomized controlled trials, case-control studies, and observational studies that have been published as full-text publications in the English language during the last five years are examples of primary research studies.

Exclusion Criteria

Excluded are studies involving patients who do not have a confirmed diagnosis of IBD, studies that do not provide information on p-ANCA assessment in IBD patients, studies that do not report relevant outcomes related to IBD diagnosis or management, studies conducted on animals rather than human subjects, studies published in languages other than English, unless English translations are available, studies published before the year 2018, and studies based on grey literature.

Quality Assessment

This systematic review included observational studies, non-randomized clinical trials, and cohort studies. Quality appraisal tools such as the Joanna Briggs Institute (JBI) Check tool and Newcastle-Ottawa were applied to assess the risk of bias during the selection of papers. Only those articles that met more than 70% of the criteria were chosen. Table 4 displays the caliber of the chosen papers.

**Table 4:** Quality assessment JBI: Joanna Briggs Institute

Author	Type of study	Quality tool appraisal	1	2	3	4	5	6	7	8	9
Torres et al. [4]	Case-control study	Newcastle-Ottawa	⁺	⁺	⁻	⁺	⁺	⁺	?	?	⁺
Imakiire et al. [6]	Case-control study	Newcastle-Ottawa	⁺	⁻	⁺	⁺	⁺	⁺	⁻	⁺	⁻
Horn et al. [1]	Cohort study	Newcastle-Ottawa	⁺	⁺	⁺	⁺	⁻	⁺	⁺	?	⁺
Yoshida et al. [10]	Cohort study	Newcastle-Ottawa	⁺	⁺	⁻	⁺	⁺	⁺	⁻	⁺	⁻
Laass et al. [7]	Cohort study	Newcastle-Ottawa	⁺	⁺	⁺	?	⁺	⁻	⁺	⁻	⁺
Aoyama et al. [5]	Cohort study	Newcastle-Ottawa	⁺	⁺	⁻	⁺	?	⁺	?	⁻	⁺
Mizuochi et al. [3]	Cohort study	Newcastle-Ottawa	⁺	⁺	?	⁺	⁺	?	⁺	⁺	⁺
Gao and Zhang [9]	Cohort study	Newcastle-Ottawa	⁺	⁺	⁻	⁺	⁺	⁺	⁺	⁻	⁺
Lee et al. [8]	Cross-sectional	JBI	⁺	⁺	⁺	⁻	?	⁺	⁻	⁺	⁺
Xu et al. [2]	Non-randomized clinical trial	JBI	⁺	⁺	⁺	?	⁺	⁺	⁺	⁻	⁻

Results

**Figure 1:**
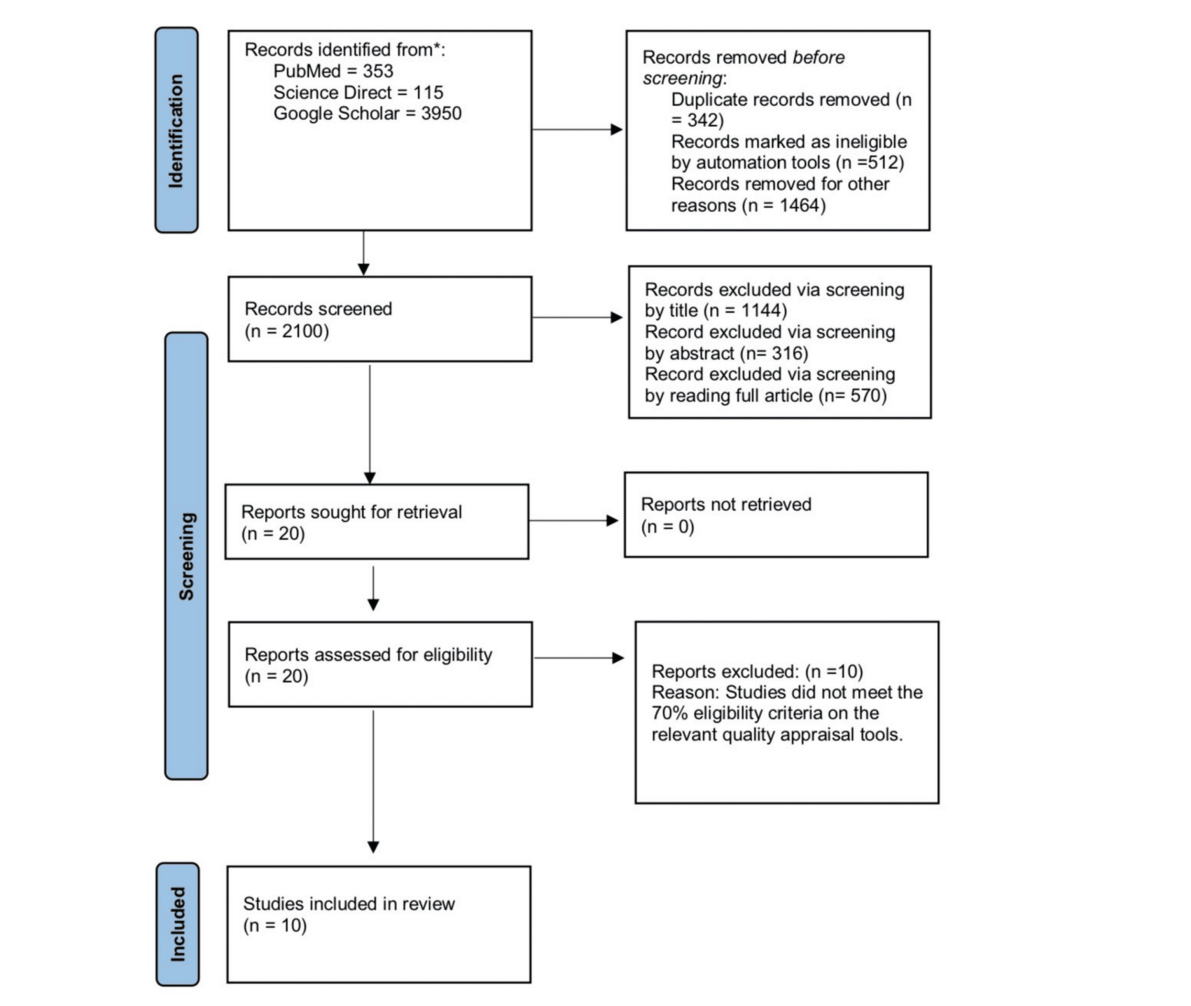
PRISMA flowchart PRISMA: Preferred Reporting Items for Systematic Reviews and Meta-Analyses

To find pertinent studies, we electronically searched four databases. At first, 4418 articles about our subject were located. Automation tools then eliminated 512 papers and 342 duplicates due to ineligibility. After screening according to inclusion/exclusion criteria and taking into account pertinent title, abstract, and full-text reading, this number was further decreased to 20. Lastly, the quality assessment tools evaluated the bias in the research. In the end, we decided to finalize 10 articles and eliminate the other 10 because they were of low quality. The search approach utilized to carry out this review is displayed in a PRISMA flowchart in Figure 1.

The summary of the final 10 articles is displayed in Table 5.

**Table 5:** Summary of final articles UC: ulcerative colitis; CD: Crohn's disease; PR3-ANCA: antineutrophil cytoplasmic antibodies specific for proteinase-3; ANCA: antineutrophil cytoplasmic antibodies; IBD: inflammatory bowel disease; MPO-ANCA: myeloperoxidase-associated antineutrophil cytoplasmic antibodies; TNF-α: tumor necrosis factor alpha; ASCA: anti-*Saccharomyces cerevisiae* antibody; CRP: C-reactive protein; PSC: primary sclerosing cholangitis

Author and year of publication	Purpose of study	Number of patients	Type of study	Result	Conclusion
Horn et al., 2018 [1]	To evaluate the diagnostic utility of PR3-ANCA for UC and the value of an antibody panel incorporating PR3-ANCA to differentiate between CD and UC	122	Cohort	PR3-ANCA was specifically associated with UC	PR3-ANCA is highly specific for UC
Lee et al., 2019 [8]	To investigate the distribution of different ANCA subtypes for IBD patients, the temporal change of ANCA status, and the predictive value of ANCA for PSC	2550	Cross-sectional	>80% of the IBD patients in our cohort who underwent ANCA testing had a positive ANCA result, and a significant proportion had positive PR3 antibodies	There is little value in ordering an ANCA for patients with IBD
Xu et al., 2020 [2]	To explore the utility of PR3-ANCA in the diagnosis of Chinese patients with IBD	216	Non-randomized	Patients with UC had higher serum PR3-ANCA levels than patients with CD or colorectal polyps	In Chinese patients with IBD, serum PR3-ANCA is a potentially helpful clinical biomarker
Mizuochi et al., 2021 [3]	To evaluate the usefulness of PR3-ANCA for the diagnosis of UC in Japanese pediatric practice	367	Cohort	PR3-ANCA showed significantly greater sensitivity (64.9%) than MPO-ANCA and good specificity	When used as a serologic diagnostic for UC diagnosis, PR3-ANCA outperformed MPO-ANCA
Torres et al., 2020 [4]	To identify serum biomarkers of CD and UC that can be detected and quantified before diagnosis	600	Case-control	Identified a panel of 51 protein biomarkers that were predictive of CD within five years	Identified a panel of serum antibodies and proteins that were predictive of patients who will receive a diagnosis of CD within five years with high accuracy
Yoshida et al., 2021 [10]	To examine whether PR3-ANCA can predict PNR to anti-TNF-α in UC patients	50	Cohort	PNR to anti-TNF-α drugs was linked to PR3-ANCA positivity	In individuals with UC, PR3-ANCA positivity can predict PNR to anti-TNF-α medications
Gao and Zhang, 2021 [9]	To explore the role of serological markers in the diagnosis of CD at an IBD referral center	196	Cohort	ASCA was a relatively specific marker for CD	ASCA was found to be the most accurate serological marker for the differential diagnosis of CD
Aoyama et al., 2021 [5]	To investigate whether PR3-ANCA levels could also predict the success of induction therapy and to compare its performance against other markers, including serum CRP and fecal hemoglobin	173	Cohort	The PR3-ANCA positivity of steroid treatment non-responders was notably greater than that of responders	In patients with moderate to severe UC, PR3-ANCA not only acts as a marker of disease activity but also foretells the side effects of steroid therapy
Imakiire et al., 2022 [6]	To investigate the clinical roles of PR3-ANCAs in the disease severity, disease extension, and clinical course of UC	173	Case-control	Patients who are positive for PR3-ANCA are found to have more progressive and severe disease	In addition to aiding in the diagnosis of UC, PR3-ANCA measurement can be used to assess the extent and severity of the disease as well as forecast its clinical trajectory
Laass et al., 2022 [7]	To investigate ANCA and PR3-ANCA in pediatric IBD	490	Cohort	PR3-ANCA-positive UC patients were characterized by more extensive disease	PR3-ANCA as a potential serological marker for pediatric UC and PSC

Discussion

p-ANCA and UC

The process of diagnosing IBD and classifying the specific subtype for individual patients poses ongoing challenges due to the intricate nature of the disease, but the presence of ANCA was significantly seen in patients with UC in a cohort study done in pediatric and adolescent patients [1,12-14]. High titers of p-ANCA were seen in patients with active UC [6,14]. According to a study, p-ANCA can predict UC patients' early clinical response to anti-TNF-α medications, and patients who tested negative for p-ANCA had a response rate to anti-TNF-α agents that was nearly twice as high as those who tested positive. However, when IIF is used to evaluate p-ANCA, it can occasionally be difficult to tell if a result is positive or negative. As a result, judgement frequently varies based on measurement facilities. So, PR3-ANCA is used instead of the p-ANCA measurement, which is c-ANCA, in other words, that is measured using enzyme-linked immunosorbent assay (ELISA) and chemiluminescence detection (CLEIA). So, in clinical practice, PR3-ANCA is a more powerful indicator of steroid use for the management of patients with UC. But the study did not tell about the course of the disease and how strong the implication of p-ANCA with UC, how that affects the management of the patient with UC, or for how long the therapy is necessary for the patient and how that impacts the life of the patient on a daily basis [10,15]. Additionally, some research indicates that biomarkers may not be adequate in determining the severity of an IBD patient's condition or predicting how well the patient would respond to treatment [6,16,17]. According to a 2019 study, ANCA positivity rates are high in IBD patients, which is concerning because ANCA is linked to systemic vasculitis. Because ANCA vasculitis and IBD can co-occur in rare circumstances, the presence of myeloperoxidase (MPO) or p-ANCA antibodies in IBD patients may prompt numerous studies aimed at ruling out systemic vasculitis. However, there is evidence in some other studies that ANCA status can change over time in patients with IBD. Furthermore, in the study cohort, PR3 positivity did not predict the specific type of IBD or the clinical course of the disease, giving us no information for patient management [8,14,18]. A study conducted on IBD patients in Japan also concluded that there is a great association between PR3-ANCA and IBD in the pediatric population. Although p-ANCA is used as a screening tool for UC, p-ANCA is considered less useful in Japanese patients [3]. While it is useful in Chinese patients, the study did not identify any association between IBD-related therapeutic drugs and PR3-ANCA levels. The study stated that PR3-ANCA may help strengthen the management of patients with IBD. There are some studies suggesting that the levels of ANCA decrease after proctocolectomy in the majority of patients with UC. Also, the drugs unconnected to IBD, such as propylthiouracil (PTU) prescribed for hyperthyroidism, can increase the levels of PR3-ANCA. So, the history of other comorbidities should be taken into consideration [2]. There is no single marker in UC that could define the preclinical phase of UC. Even p-ANCA which is the most specific marker of UC has suboptimal performance, especially in the preclinical phase of the disease, so there is no evidence of any intervention that could be of use [4,16,19-23]. There have been multiple interventions for IBD so ANCA status change cannot be attributed to one particular intervention [8,24,25]. From all the studies cited above, there is a greater association between PR3-ANCA and UC than p-ANCA, but the management association is not clear to us. In summary, all these studies stated that there is a strong association between p-ANCA and IBD, but there is much less or no evidence at all about the impact of this association on the management of patients with UC or IBD in general [26,27]. More studies should be performed to collect evidence of the implications on the management of the patients and how the strategy of the treatment should be changed, keeping in mind the positivity of ANCA in IBD patients.

p-ANCA and CD

In a study done on Chinese patients with IBD, PR3-ANCA can differentiate between CD and UC [2]. In a cohort study done in pediatric and adolescent patients, they accepted ASCA as a highly sensitive marker for CD [1,13]. Moreover, ASCA positivity is considered a more predictive factor for UC than ANCA. A study done in the pediatric population states that ASCA positivity in CD patients is associated with ileocolonic disease and may predict a more complex and severe disease course [1,26,28]. A study found high sensitivity and low specificity for the diagnosis of CD (ASCA+/ANCA-). For the management of CD, it is necessary to figure out the association between CD phenotypes and serological markers. Some studies suggested that in severe phenotypes of CD, the levels of ASCA were raised [9,19]. ASCA is the most precise serological marker for differentiating between CD and suspected CD patients. A combination of various serological markers such as ASCA, AYMA, AYCA, and FI2Y improved the accuracy of the diagnosis of CD unlike p-ANCA and PR3-ANCA in UC. It also suggested that the combined use of the serological markers used in IBD helped in the exclusion of the disease rather than making the diagnosis of CD [9,29,30]. IBD starts with a preclinical phase that is of unknown duration, but it is thought that if the preclinical phase is determined, the interventions for CD could be started earlier, which could be of benefit, but the same is not the case for UC. There is no evidence yet of which interventions could be of use to start in the preclinical phase of the disease. This study also states that when the diagnosis is made, it becomes difficult to differentiate between the pathways that are responsible for the causation of the disease and those that dysregulate the inflammatory cycle. Some therapies could be used in the preclinical phase, such as microbiome-directed therapies, antibiotic use, or dietary interventions [4,28]. The p-ANCA positivity is present in 1/3 or more patients with UC but is only present in a few patients with CD. So, this differentiates between UC and CD. There is a high prevalence of PR3-ANCA positivity in UC and CD, so there is more disease severity in patients suffering from UC than in patients suffering from CD. There is evidence that with treatment modalities, the levels of p-ANCA decrease, but how much decrease is present with specific interventions is not yet known [2,30]. No single marker or combination of markers provides good predictive performance in patients with UC in contrast to CD. If the markers were predicted before the onset of the symptoms, it would lead to the early initiation of patient management and prevent complications such as vasculitis in patients suffering from CD [4]. None of the studies talked about the management of patients with IBD or how these serological markers play a role in the treatment of IBD though there is a strong association between ASCA and IBD. To see the implications of these serological markers on the treatment of the patients, more studies are needed to be performed, and how the management of the patients should be improved should also be studied. The same goes for which interventions cause a decrease in the p-ANCA levels and how much decrease is caused by the drugs.

Limitations

The majority of the papers in this systematic review are observational studies. Articles that were not published within the last five years or that were not written in English were not included. Consequently, it may result in the omission of some pertinent studies. Moreover, we should consider the incomplete nature of the data obtained from the studies, which further contributes to the potential uncertainties in our conclusions. There were fewer studies available on the treatment aspect in relation to p-ANCA. Limited papers on the interested topic were available in the last five years.

## Conclusions

This systematic review suggests that there is a stronger association between p-ANCA and UC than CD in patients with IBD in patients with all age groups. Moreover, many studies have implicated a decrease in p-ANCA to some extent with medical or surgical interventions, but the exact intervention is not yet clear. We now know that p-ANCA and IBD are significantly correlated, so managing IBD in relation to p-ANCA should be our next priority. Studies must further evaluate which specific interventions cause a decrease in p-ANCA and how much time they may help reduce the complications of IBD.
