# Correction: Inflammatory Bowel Disease and Its Association With Perinuclear Antineutrophil Cytoplasmic Antibodies: A Systematic Review

**DOI:** 10.7759/cureus.c326

**Published:** 2025-09-26

**Authors:** Harleen Kaur, Malik Kasapoglu, Rajesh Yadavalli, Sarosh Nawaz, Abdulaziz Althwanay, Esraa M AlEdani, Ann Kashmer Yu

**Affiliations:** 1 Department of Internal Medicine, California Institute of Behavioral Neurosciences & Psychology, Fairfield, USA; 2 Department of Internal Medicine, Rajiv Gandhi Institute of Medical Sciences, Adilabad, IND; 3 Department of Psychiatry, California Institute of Behavioral Neurosciences & Psychology, Fairfield, USA; 4 Department of Dermatology, California Institute of Behavioral Neurosciences & Psychology, Fairfield, USA; 5 College of Medicine, Imam Abdulrahman Bin Faisal University, Dammam, SAU; 6 Department of Dermatology and Internal Medicine, California Institute of Behavioral Neurosciences & Psychology, Fairfield, USA

This article has been corrected to fix the following typo in the first paragraph of the Results section: "In the end, we decided to finalize 10 articles and eliminate the other **9** because they were of low quality." corrected to "In the end, we decided to finalize 10 articles and eliminate the other **10 **because they were of low quality."

